# Mismatch Repair Deficiency and the Role of Non-Canonical Functions in Cancer: Diagnosis and Therapeutic Implications

**DOI:** 10.3390/ijms26199312

**Published:** 2025-09-24

**Authors:** Alicja Dąbrowska, Jakub Mastalerz, Zofia Łapińska, Iwona Deszcz, Agnieszka Chwiłkowska, Nina Rembiałkowska

**Affiliations:** 1 Faculty of Medicine, Wroclaw Medical University, Pasteura 1, 50-367 Wroclaw, Poland; alicja@dabrowski.wroclaw.pl (A.D.); jakub.mastalerz18@gmail.com (J.M.); 2 Department of Molecular and Cellular Biology, Faculty of Pharmacy, Wroclaw Medical University, Borowska 211A, 50-556 Wroclaw, Poland; zofia.lapinska@umw.edu.pl (Z.Ł.); agnieszka.chwilkowska@umw.edu.pl (A.C.); 3 Division of Immunopathology and Molecular Biology, Department of Basic Medical Sciences and Immunology, Faculty of Pharmacy, Wroclaw Medical University, Borowska 211A, 50-556 Wroclaw, Poland; iwona.deszcz@umw.edu.pl

**Keywords:** DNA mismatch repair, MMR molecular mechanisms, dMMR cancers, drug therapy, temozolomide, mutation

## Abstract

The mismatch repair system is critical for correcting base–base mismatches and insertion-deletion loops during DNA replication. Deficiencies in MMR (due to mutations in MLH1, MSH2, MSH6, or PMS2) lead to microsatellite instability and contribute to the development of various cancers, such as Lynch syndrome-related colorectal cancer and sporadic tumors. This review will delve into the molecular basis of MMR deficiency. Additionally, the review will cover diagnostic approaches for detecting MSI and MMR deficiency, including next-generation sequencing and PCR-based methods. The implications for treatment will be discussed, emphasizing immune checkpoint inhibitors (e.g., pembrolizumab) that target tumors with high mutational burdens due to MMR deficiency, as well as novel therapeutic approaches like synthetic lethality exploiting DNA repair vulnerabilities.

## 1. Methodology

A literature search was done on PubMed and EMBASE using the keywords “DNA mismatch repair”, “MMR molecular mechanisms”, “MMR neuroendocrine cancer”, “MMR sarcoma”, “MMR heterogeneity”, “MMR liquid biopsy”, “MMR ctDNA”, “MMR functional assays”, “MMR altering therapies”, “dMMR”, and “pMMR”. The reference lists of the found studies were also reviewed. We included full-text articles in the English language. Review articles and observational studies were analyzed for our review; however, case studies were excluded. The time range of the articles included was 2019–2024, unless older articles thoroughly described well-known mechanisms in MMR.

## 2. Introduction

DNA mismatch repair (MMR) is a crucial mechanism that corrects DNA replication errors and maintains genome stability. It involves a complex of proteins: MutS homologs (MSH2, MSH3, MSH6) and MutL homologs (MLH1, PMS2), which recognize and repair mismatched base pairs [1]. The process involves DNA binding, strand excision, and resynthesis by DNA polymerase. MMR is also involved in non-canonical functions, such as oxidative DNA damage repair and immunoglobulin diversification. Defects in MMR genes can lead to microsatellite instability (MSI), a hallmark of several cancers, particularly solid tumors [2].

One of the most common methods used to evaluate MMR status is immunohistochemistry (IHC)—a laboratory technique using antibodies [3]. In the context of MMR assessment, IHC can usually examine proteins such as MSH2, MSH6, MLH1, and PMS2. A lack of expression of these proteins indicates MMR deficiency, a hint of probable MSI occurrence, and a predictor for response to immunotherapy. Other assays for MMR status include PCR-based methods like the pentaplex MSI assay and next-generation sequencing (NGS), which detect MSI directly. These techniques are often complementary to IHC [4,5]. Furthermore, ctDNA is becoming increasingly popular in evaluating the MMR status in cancer patients, as it might offer better results, especially when the amount of tissue samples is limited [6].

MMR deficiency has also been implicated in rarer and understudied cancers, such as sarcomas, neuroendocrine tumors, and hematological malignancies. In sarcomas, MSI may influence tumor behavior and response to treatments, although this is less well explored compared to other cancer types [7]. Similarly, in hematological cancers, such as lymphomas, MMR defects can contribute to genomic instability, impacting tumor progression and prognosis. Research in these areas is still emerging, but early studies suggest that MMR deficiency may provide diagnostic and therapeutic insights, particularly in immunotherapy, where dMMR cancers are more responsive to checkpoint inhibitors [8]. However, more research is essential to understand the role of MMR in these less-studied cancers and achieve better treatment results. In our study, we will discuss the role of MMR in cancer diagnosis and as a biomarker. Furthermore, we will describe the novel potential treatment targets.

## 3. MMR Pathway in Normal Human Cells

The human MMR system is a sophisticated and highly conserved DNA repair mechanism responsible for maintaining genomic stability by correcting replication errors. This system primarily resolves base–base mismatches and small insertion-deletion loops (IDLs) that arise during DNA replication. By ensuring replication fidelity, MMR prevents the accumulation of mutations that could otherwise contribute to genomic instability, a hallmark of cancer and other diseases [9]. The MMR process involves multiple protein complexes that sequentially detect, excise, and repair mismatched regions of DNA. Below, the molecular mechanisms of human MMR are described in detail, beginning with mismatch recognition and concluding with repair completion. Table 1 summarizes the main proteins involved in this pathway and their primary functions.

The first step in MMR is the recognition of replication errors by MutS homolog (MSH) proteins. Two principal heterodimers are responsible for this recognition: MSH2-MSH6 (MutSα) and MSH2-MSH3 (MutSβ) [9]. MutSα is specialized for detecting single base–base mismatches and small IDLs, whereas MutSβ primarily identifies larger IDLs. MSH2 serves as the scaffold protein in both complexes, stabilizing the heterodimer and facilitating its binding to the DNA. When a mismatch is encountered, the MutSα or MutSβ complex binds to the site with high specificity, forming a clamp around the DNA helix [10]. This binding event induces a conformational change in the complex, driven by ATP binding, which recruits downstream repair factors to the site of damage.

Once the mismatch is recognized, MutL homolog (MLH) proteins are recruited to initiate the excision phase of repair. The primary heterodimer involved in this step is MLH1-PMS2 (MutLα), the main endonuclease in human MMR [11,12]. MutLα interacts with the mismatch-bound MutS complex in an ATP-dependent manner, stabilizing the repair machinery at the site. MutLα is also responsible for introducing a nick in the newly synthesized DNA strand near the mismatch. The ability to distinguish the newly synthesized strand from the parental strand is critical for repair fidelity [13]. This discrimination is facilitated by pre-existing nicks in the lagging strand during replication or, on the leading strand, by interactions with replication factors such as proliferating cell nuclear antigen (PCNA).

PCNA plays a central role in coordinating MMR [14,15]. PCNA encircles the DNA as a sliding clamp and interacts with MutLα to localize the endonuclease activity to the appropriate strand. It also serves as a platform for recruiting other repair proteins. PCNA loading onto the DNA is mediated by replication factor C (RFC), which ensures the proper assembly of the repair complex [16]. With PCNA in place, MutLα introduces a nick in the nascent strand, marking the starting point for excision of the mismatched DNA.

The excision phase of MMR is mediated by exonuclease 1 (EXO1), a 5′3′ exonuclease that removes the DNA segment containing the mismatch [9]. The activity of EXO1 is tightly regulated to prevent excessive degradation of the DNA strand [13]. As EXO1 degrades the DNA, replication protein A (RPA) binds to the single-stranded DNA to prevent secondary structure formation and protect the strand from further degradation. RPA also facilitates the recruitment of DNA synthesis machinery to fill the gap created by excision [16].

**Figure 1:**
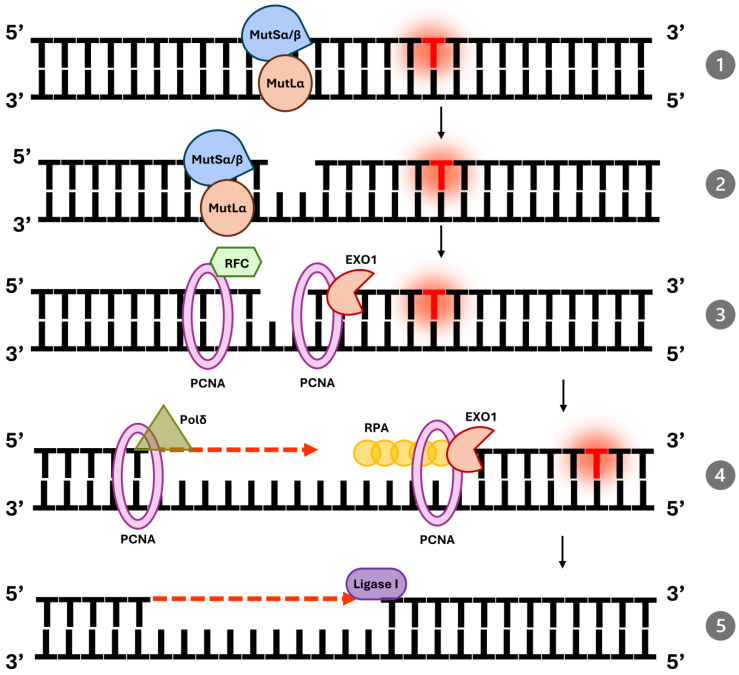
Mismatch repair pathway in human cells: (**1**,**2**) Mismatch Recognition the MutSα (MSH2-MSH6) or MutSβ (MSH2-MSH3) complexes recognize base–base mismatches or insertion–deletion loops, binding to the mismatched DNA and cleaves the DNA at a distance from the mismatch; (**3**) PCNA, loaded onto DNA by RFC, acts as a sliding clamp to facilitate the coordination of downstream repair events; excision of the Mismatched DNA Segment and Resynthesis Exonuclease 1 (EXO1), in conjunction with PCNA excises the DNA segment containing the mismatch; (**4**) Replication protein A (RPA) binds to the single-stranded DNA to protect it from further degradation and to prevent secondary structure formation. DNA polymerase δ, with the aid of PCNA, synthesizes the replacement strand using the undamaged template as a guide; (**5**) DNA Ligation DNA ligase I seals the remaining nick, completing the repair and restoring the integrity of the DNA molecule.

Following excision, DNA polymerase δ synthesizes new DNA to replace the excised region. Using the undamaged template strand as a guide, DNA polymerase δ ensures accurate resynthesis of the repaired strand. PCNA, which remains associated with the DNA throughout the repair process, enhances the processivity of DNA polymerase δ, allowing it to efficiently synthesize the entire gap [14]. After the synthesis step, the final nick in the DNA is sealed by DNA ligase I, restoring the integrity of the DNA molecule and completing the repair process [12]. Figure 1 shows the representative MMR pathway.

The next part of this review describes the critical role of MMR in cancer prevention and the consequences of its inefficiency in detail. Disturbances in MMR have wide-ranging consequences for genome integrity and therapy response, underscoring the need for a detailed molecular-level understanding of this pathway.

## 4. Emerging Molecular Mechanisms of MMR Deficiency

### 4.1. The Non-Canonical Roles of MMR Genes

Recently, there have been increasing studies regarding the non-canonical role of MMR genes. Numerous studies suggest that these genes play a crucial role in traditional DNA repair and in influencing the cells’ immune response, the hormonal balance in the organism, and the cellular stress response [9,10].

One recent study described the role of the MMR pathway in oxidative DNA damage repair. The siRNA screens showed a lethal interaction between loss of the MMR genes—MSH2 and MLH1—and inhibition of polymerase beta (POLβ) or polymerase gamma (POLγ) in the cellular models. Notably, the MSH2/POLβ synthetic lethality was associated with an accumulation of 8-oxo-G lesions in nuclear DNA, whereas the MLH1/POLγ led to a buildup of mitochondrial 8-oxo-G lesions [11,12]. Furthermore, Martin et al. [13] found out that silencing PTEN-induced putative kinase 1 (PINK1) was synthetically lethal in MMR-deficient cell lines, as it led to reactive oxygen species (ROS) levels and a subsequent accumulation of oxidative DNA lesions in both the nucleus and mitochondria [13]. Those findings suggest that inhibition of POLβ, POLγ, or PINK1 in cells with functional MMR leads to the formation of 8-oxo-G lesions, which are eventually repaired by the MMR pathway or in collaboration with it.

Furthermore, MMR is shown to be crucial in the DNA demethylation (DDM) process. It is now well-known that long-patch DNA excision and resynthesis repair pathways involve long-patch Base Excision Repair (BER) and MMR mechanisms. It has been shown that the deamination products of cytosines, such as dU•G, 5ohU•G, T•G, and 5hmU•G mismatches, might serve as potential triggers for Active DNA Demethylation (ADDM) [14]. Notably, these mismatches can be substrates for both MMR and long-patch BER pathways, with MMR having a more extensive repair range. During specific stages of active demethylation in mouse zygotes, activation-induced deaminase (AID) mediated cytosine deamination coupled with UNG2-initiated long-patch BER might play a significant role. It is well-established that cytosine deamination and the interaction between BER and MMR pathways are essential for somatic hypermutation (SHM) in the immunoglobulin locus of activated B cells. However, the specific contributions of these pathways to genome-wide or locus-specific ADDM still need further research [14,15].

Grin et al. [16] conducted a study where the roles of short-patch and long-patch BER, along with non-canonical MMR pathways, were assessed. The repair of deaminated and oxidized cytosine derivatives and the non-targeted erasure of 5mC marks were investigated. They found that the repair was efficient and accompanied by the removal of adjacent and more distant 5mC residues in cell extracts from Mouse Embryonic Fibroblasts (MEF) and BL2 cells. Moreover, the presence of just two lesion-containing mismatches in closed circular DNA was sufficient to trigger MMR-dependent lesion repair and localized demethylation of plasmid DNA, achieving approximately 23% erasure of 5mC in constructs with hmU-15-mC-14-hmU sites in MEF extracts [16].

Interestingly, MMR plays a role in suppressing mutagenesis induced by ultraviolet (UV) light and dietary compounds. The MMR heterodimer MutSα is recruited to sites of localized UV damage and then binds mismatched nucleotides to opposite photolesions. It has been suggested that a non-canonical MMR pathway known as “post-translesion synthesis (TLS) repair” removes nucleotides that TLS incorrectly incorporated opposite the damaged bases. Post-TLS repair reduces mutagenesis caused by DNA damage while the resulting single-stranded DNA regions activate DNA damage signaling pathways [17,18].

Although first characterized in 2005, the non-canonical roles of hMSH2 in estrogen receptor signaling have only recently been placed into a therapeutic context [19]. A 2021 comprehensive review of mismatch-repair alterations in hormone receptor-positive breast cancer highlighted that dysregulation of MMR proteins—notably hMSH2—contributes to endocrine therapy resistance and may help identify patients who could benefit from combining DNA-repair inhibitors (e.g., PARP inhibitors) with standard endocrine agents [20]. Building on this, a 2024 analysis of non-repair functions of MMR components showed that hMSH2’s selective coactivation of ERα amplifies proliferative transcriptional programs in ER-driven tumors, proposing that disrupting the ER–hMSH2 interface could restore hormone-therapy sensitivity in refractory breast and endometrial cancers (EC) [19,21].

### 4.2. Alkylation Damage in Cells

Alkylation lesions in DNA and RNA may be caused by endogenous metabolites, environmental agents, and alkylating drugs. Simple methylating compounds form adducts primarily at N- and O-atoms of DNA bases, which are repaired by direct base repair or base excision. O^6^-methylguanine (O^6^-meG) has miscoding properties and thus leads to mutations. This might be prevented by O^6^-alkylguanine-DNA alkyltransferase (MGMT/AGT). However, the genotoxic and cytotoxic effects of O^6^-meG result from MMR-mediated recognition of O^6^-meG:T (or C) mispairs, triggering futile repair cycles and DNA double-strand breaks due to the checkpoint response involving ATR/CHK1 and ATM/CHK2 kinases [22,23]. What is more, DNA MMR proteins are also needed for DNA-damage signalling, which causes activation of cell cycle checkpoints and apoptosis. Furthermore, loss of either MutSa or MutLa is proven to decrease DNA-damage responses to both the SN1-type alkylating agents and 6-TG [24]. Loss of MMR function in MGMT-deficient cells results in resistance to cytotoxicity but not to mutagenesis.

### 4.3. MMR Heterogeneity

It has been proven that MMR and MSI status play a significant role in the clinical course of many cancers, especially EC [25]. In one of the studies, the authors stated that EC with subclonal or heterogeneous MMR expression is associated with a high recurrence rate, with metastatic or recurrent lesions often showing MMR deficiency. Transcriptional analysis suggests an increased chance for migration and metastasis, indicating that clonal MMR deficiency may cause tumor aggressiveness. Therefore, reporting MMR IHC without acknowledging subclonal or heterogeneous staining patterns may miss the opportunity for biomarker-guided therapy [26].

Furthermore, similar findings were made regarding metastatic colorectal cancer (mCRC), as another study exhibited MMR status heterogeneity between primary and corresponding metastatic in almost 12% of the patients. The occurrence of the heterogeneous MMR phenotype was significantly higher in primary tumors with deficient MMR (dMMR) than with proficient MMR (pMMR). Notably, patients with or without MMR status heterogeneity had similar overall survival. However, it is important to test the metastatic site, as the divergence of MMR status might potentially affect the immunotherapy [27]. In mCRC, MMR status is now recognized as a key biomarker that guides treatment selection [28]. Tumors with dMMR (usually MSI-high) respond favorably to Immune Checkpoint Inhibitors (ICI)—for example, pembrolizumab immunotherapy has demonstrated significantly longer progression-free survival than chemotherapy as first-line treatment for dMMR mCRC [29]. In contrast, pMMR (microsatellite-stable) tumors derive little or no benefit from single-agent immunotherapy [30] and are instead managed with conventional cytotoxic chemotherapy regimens, often augmented by targeted monoclonal antibodies—e.g., adding an anti-VEGF agent, or an anti-EGFR antibody in RAS wild-type cases [31]. Reflecting these differences, current treatment guidelines recommend routine testing of MMR/MSI status in mCRC and advise using immunotherapy for dMMR cases while guiding pMMR cases toward chemo ± targeted therapy [31,32].

## 5. MMR Deficiency in Understudied Cancer Types

### 5.1. Constitutional MMR Deficiency Syndrome

Constitutional mismatch repair deficiency (CMMRD) syndrome is a rare childhood cancer predisposition disorder caused by biallelic mutations in MMR genes (*MLH1, MSH2, MSH6, PMS2*). A broad spectrum of early-onset malignancies, including hematological cancers, brain tumors, and colorectal cancer, occur in CMMRD patients [33,34]. Notably, they often display features resembling neurofibromatosis type 1, such as café au lait spots, which can lead to misdiagnosis. Moreover, the syndrome is frequently underrecognized due to its variable clinical presentation and phenotypic overlap with other cancer syndromes [35,36]. Therefore, it is crucial to raise awareness among physicians about the existence and clinical relevance of this disease. Improved knowledge of clinical criteria and diagnostic methods is necessary for early identification and appropriate management of CMMRD patients and their families.

### 5.2. MMR Deficiency in Rare Solid and Hematologic Tumors

There is growing evidence and amount of studies that cover the MMR status and its role in the less common cancers, such as Neuroendocrine Cancer (NEC), sarcomas, or hematologic cancers. A recent study examined the influence of the MMR status on the overall survival of patients with neuroendocrine tumors (NET) [37]. The authors revealed that pMMR had shorter overall survival than dMMR patients. Moreover, the results of using ICI in these tumors are still conflicting. Therefore, it is important to establish a proper selection of patients undergoing ICIs treatment, in which dMMR status and PD-L1 expression seemed to be predictors of good response to treatment. Notably, it was established that dMMR status is more common in NECs, which also exhibit higher levels of intratumor lymphocytic infiltration and are associated with a better prognosis. Thus, immunological therapies should be employed and further researched in a subset of NEC [38]. On the other hand, another study suggests that the risk of progression of pancreatic NET does not correlate with the dMMR. According to the evaluation criteria for MMR defects, none of the pancreatic NETs showed a nuclear staining loss for MSH2, MSH6, MLH1, and PMS2 [39].

Tanaka et al. [40] conducted a study examining chromosome translocation in Ewing sarcoma cells. Sister chromatid exchange (SCE) reflects the improper resolution of Holliday junctions during homologous recombination in S-phase cells. In a study treating cells with EdU to induce fork stress, SCE was most observed in MMR-deficient DLD-1 cells, suggesting heightened HR activity. This contrasts with MMR-proficient SW620 cells, which exhibited less frequent SCE, highlighting the role of MMR deficiency in HR regulation [40]. Clinically, MMR deficiency—and its associated increase in HR—is a hallmark of microsatellite-instable tumors and serves as a predictive biomarker for PARP inhibitor sensitivity (e.g., olaparib) and for responsiveness to immune checkpoint therapies in MSI-high cancers, as evaluated in ongoing trials such as #NCT02484404.

Although MMR is known to be a predictive biomarker in numerous cancers, there are not many studies regarding synovial sarcomas (SS). In one of the recent studies, MMR status (MLH 1, MSH2, MSH6, and PMS2) and its association with immunotherapy results were assessed in SS patients. Most of the SS cancers express low Tumor Mutational Burden (TMB). However, those occasional tumors with highly expressed TMB respond well to checkpoint inhibitor therapy [41]. Given its clinical relevance, TMB assessment should be considered in treatment planning. However, it should be noted that MMR status determined by IHC fails to accurately identify high-TMB patients. On the other hand, a combined analysis of TMB, MMR protein status, and immunohistochemical evaluation of PD-L1 and PD-1 expression was the most effective strategy for identifying patients who could respond favorably to checkpoint immunotherapy [41]. These findings are in line with clinical evidence from broader soft-tissue sarcoma cohorts, in which pembrolizumab demonstrated meaningful activity in selected patients, underscoring the potential of immunotherapy in this group of tumors [42].

The status of MSI and MMR deficiency in hematologic malignancies remains largely undefined. However, MMR deficiency is observed in about 9% of classical Hodgkin Lymphoma (cHL), 8% of Primary Mediastinal B-cell Lymphoma (PMBCL), and 0.3% to 3.2% of Diffuse Large B-cell Lymphoma (DLBCL) [43,44]. The link between MSI/MMR deficiency and response to checkpoint inhibitors in lymphoma is unclear, as these cancers were not included in MSI-H/dMMR immunotherapy trials [45]. Additionally, one of the studies stated that Type A MSI was strongly connected with poor clinical outcomes in ATLL patients [46]. Therefore, the role of MSI as a predictive and prognostic biomarker in hematological malignancies should be considered and further investigated.

## 6. Novel Diagnostic Approaches for MMR Deficiency

### 6.1. Liquid Biopsy and ctDNA

The standard approach for classifying MSI/dMMR tumors typically involves IHC or PCR-based assays targeting a panel of five microsatellite regions. However, new molecular methods utilizing tumor tissue or plasma samples are emerging. These advancements may offer more sensitive and less invasive options for identifying MSI status, expanding the capabilities of current diagnostic practices [47].

Circulating tumor DNA (ctDNA) can diagnose MSI in routine clinical practice, mainly when tissue samples are limited. This minimally invasive method allows repeated testing to monitor disease in real-time based on ctDNA kinetics [48]. Studies show that changes in MSI levels during ICI treatment correlate with tumor response and can predict outcomes earlier than traditional imaging [49]. Furthermore, ctDNA analysis can identify somatic MSI in patients initially diagnosed with microsatellite stable (MSS) tumors, supporting its potential for personalized cancer management [6,50].

Lynch syndrome (LS) is an autosomal dominant genetic disorder characterized by germline mutations in MMR genes, particularly *MLH1*, *MSH2*, *MSH6*, *PMS2*, and *EPCAM*. Diagnosis involves clinical criteria, IHC for MMR protein expression, and molecular tests for MSI [51,52].

Boeri et al. [53] conducted a study that retrospectively examined LS patients using a new multiplex drop-off digital polymerase chain reaction (dPCR) assay, reaching mutant allele frequencies (MAFs). Patients with lesions exhibited higher MAFs for BAT26, BAT25, and NR24 compared to controls, though these differences were unrelated to dysplasia severity or other pathological features. When the three markers were jointly evaluated to determine blood-based MSI status, the resulting analysis achieved an AUC of 0.80, enabling reliable discrimination between individuals with and without lesions [53].

The method using ctDNA might help diagnose MSI in gliomas. However, in gliomas, low levels of ctDNA are observed; consequently, methods with high sensitivity are needed—one of the studies sequenced plasma samples with CAPP-seq (AVENIO) and tissue samples with TSO500. Glioma-derived ctDNA mutations were found in more than 93% of plasma samples. Mutations of MSH2 and MSH6 were the most common circulating gene alterations after temozolomide (TMZ) treatment. Notably, they were often observed to appear in plasma before appearing in tumor tissue at the time of surgery for recurrence. Thus, plasma ctDNA detection of new MMR gene mutations do not present in the initial tissue biopsy might provide an early indication of the development of chemotherapy resistance [54].

However, further extensive cohort studies of all the novel methods and cost-effectiveness analyses should be performed before their implementation in clinical practice.

### 6.2. Functional Assays for MMR Activity

Next-generation sequencing-based MSI detection is widely used to assess MSI. However, MSI tumors detected using NGS and their relevance to MSI-PCR and mismatch repair deficiency (dMMR) are unclear. The cut-off value of MSI-H and MSS using NGS remains controversial and requires further clarification [55].

On the other hand, Gilson et al. [56] conducted a study in which they evaluated three molecular-based assays for MSI detection in colorectal and endometrial patients: a custom capture-based NGS panel, the Idylla MSI system, and the Bio-Rad ddPCR MSI assay. When compared with established MSI testing methods, these approaches demonstrated strong concordance, with agreement rates of 93.3% for the NGS panel and 100% for both Idylla and Bio-Rad ddPCR. Interestingly, these molecular methods remained effective even with FFPE material of suboptimal DNA quality and were able to clarify MSI status in cases where IHC results were indeterminate, such as confirming MSS in ambiguous cases. Consequently, authors showed the importance of combining IHC with molecular assays for accurate MMR/MSI testing to optimize immunotherapy decisions [56]. Smithgall et al. [57] performed another study regarding the assessment of the NGS panel. They found a 98% (322/328) concordance with the MSI and IHC results. Out of the discrepant cases, most exhibited dMMR with MSS, and one showed pMMR with MSI-H. Thus, these results emphasize the importance of selecting the appropriate tumor tissue for IHC and molecular testing and show that NGS might help resolve discrepant MMR and MSI results [57].

A distinct, potentially important functional assay is the Methylation Tolerance-Based Functional Assay, which might help evaluate the MMR gene variants of unknown significance (VUS). This assay measured cell response to the cytotoxic methylating agent and thus identified patients with constitutional dMMR syndrome. MMR status influences cellular responses to various chemotherapeutic agents, including TMZ, 6-thioguanine, and cisplatin, as the MMR-proficient cells exhibit cell cycle arrest and apoptosis upon treatment with methylating agents [58,59]. Comparing the results with prediction scores confirmed that it may be used to discriminate VUS affecting the gene functions from those that did not. Such assays may be used to personalize the treatment method and provide better results and overall survival [60].

To facilitate clinical decision-making, Table 2 summarizes the diagnostic workflow of MMR/MSI testing and outlines its therapeutic implications, serving as a practical guide for personalized cancer treatment.

## 7. MMR Altering Therapies

Defects in MMR might lead to genome-wide mutations, including mutations in protein-encoding DNA. These mutated peptides are packed into proteasomes and loaded into the MHC-I complex, which results in high immunogenicity in the dMMR tumors [61]. Therefore, dMMR metastatic colorectal cancers (mCRC) are responsive to immunotherapy. In clinical trials, combining the anti-PD-1 drug, nivolumab, with the anti-CTLA4 drug, ipilimumab, leads to a significantly better objective response rate and disease control rate [62].

However, the vast majority of mCRC is pMMR and, therefore, does not respond to immunotherapy with ICI [63].

Temozolomide is an alkylating agent used as a treatment option for many solid tumors [64]. It causes DNA damage primarily by methylating the O6 position of guanine, which can further lead to mispairing with thymine. The MMR system recognizes the O6-methylguanine-thymine mismatches; however, it cannot reverse the O6-methylguanine lesion, causing the cycle of futile repairs and, eventually, cell apoptosis [65].

A group of researchers has demonstrated that the use of TMZ in preclinical models of CRC leads to MMR deficiency, as well as sensitization to immunotherapy [66]. The same group designed a clinical trial where MMR-proficient, RAS-mutant mCRC patients received priming therapy with TMZ [67]. This treatment caused a mutation of the MMR gene MSH6, which was detected in 94% of patients. Those patients gained susceptibility to immunotherapy with pembrolizumab and achieved disease stabilization after treatment with this drug [67]. Interestingly, the majority of TMZ-sensitive cell lines exhibited diminished O-6-methylguanine DNA Methyltransferase (MGMT) expression, consistent with the established role of MGMT as the principal enzyme mediating the removal of TMZ-induced DNA adducts [66]. The authors revealed that TMZ-induced resistance through MMR abrogation led to an elevated mutational burden, sustained neoantigen generation in human colorectal carcinomas, and activation of immune surveillance in murine models. These findings imply that disrupting DNA repair pathways can amplify the neoantigen repertoire of tumor cells.

**Figure 2:**
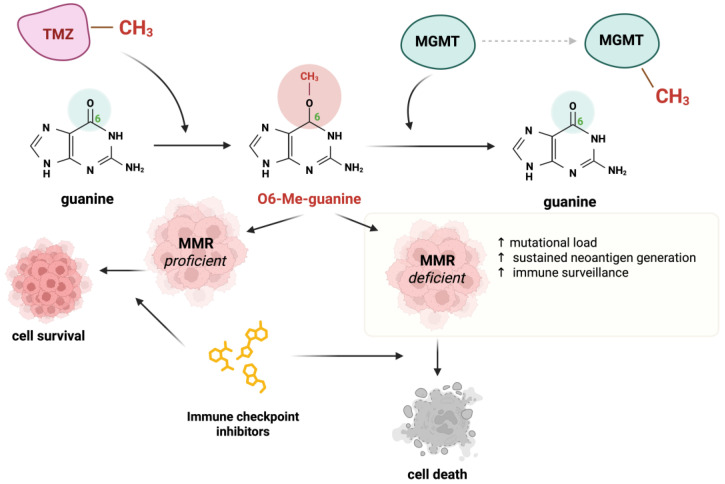
The mechanisms of action of temozolomide (TMZ). DNA mismatch repair (MMR) proficiency promotes cytotoxicity, while O-6-methylguanine DNA Methyltransferase (MGMT) activity counteracts DNA damage. While MMR deficiency increases mutational load and neoantigen renewal, enhancing responsiveness to immune checkpoint inhibitors.

In another study, another group of researchers used TMZ on five human melanoma cell lines: Mel18, A375, WM266-4, G361, and TXM18. Unfortunately, the TMZ increased the total mutation burden in those cell lines, but the MSI due to dMMR was not observed in any of the examined cell lines [68]. The mechanisms of action of TMZ have been presented in Figure 2.

In 2023, it was proven that DNA polymerase β (POLB), a key component of the BER pathway, is essential for the survival of dMMR cells and their resistance to thiopurine. POLB treatment with its inhibitor, oleanolic acid, induced synthetic lethality in the context of dMMR by increasing apurinic/apyrimidinic sites, DNA strand breaks, and apoptosis [69].

**Figure 3:**
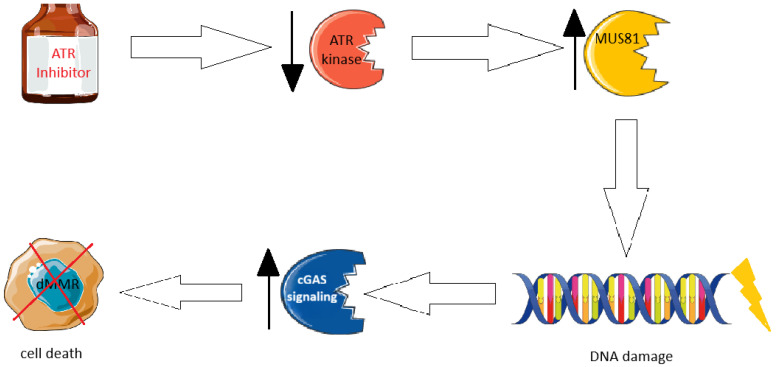
ATR inhibitors are one of the promising therapies in dMMR cancer treatment.

The ATR checkpoint kinase is central in regulating the DNA replication stress response and is essential for maintaining replication fork stability [70]. Several MMR proteins are substrates of ATR. It was found that the inhibition of ATR kinase selectively kills dMMR cancer cells by inducing MUS81 nuclease- and replication-dependent DNA damage, leading to increased cytosolic DNA fragments and activation of cGAS signaling. Those antitumor effects are partially associated with CD + T cells. It was also shown that in the syngeneic mouse models, combining ATR inhibitors with anti-PD-1 antibodies more effectively suppresses the growth of MMR-deficient tumors than using either ATR inhibitors or anti-PD-1 antibodies alone [71]. In another mouse model, it was stated a combination of ATR inhibitor and irradiation enhanced CD8T cell infiltration, as well as the efficacy of anti-PDL1 therapy in CRC [72]. The ATR inhibitors’ action mechanism has been presented in Figure 3.

Two recent phase II trials have provided compelling clinical evidence that TMZ priming can convert microsatellite-stable colorectal cancers into tumors susceptible to immune checkpoint blockade. In the single-arm ARETHUSA study (#NCT03519412), 30 patients with refractory, MGMT-silenced, RAS-mutant metastatic CRC received induction TMZ until disease progression; those whose tumors acquired a hypermutator phenotype (defined by a rise in TMB above 20 mutations/Mb) were subsequently treated with pembrolizumab [67]. TMZ exposure reproducibly induced inactivating mutations in the MMR gene MSH6 in the majority of evaluable cases, and this emergent hypermutation was detectable both in tumor biopsies and ctDNA. Among the first six patients who met criteria for checkpoint therapy, four achieved prolonged disease stabilization, thereby demonstrating that TMZ-driven MMR disruption can generate sufficient neoantigen burden to elicit anti-PD-1–mediated control in a subset of previously immunotherapy-resistant CRCs.

The subsequent multicenter MAYA trial (#NCT03832621) expanded on these findings by combining TMZ priming with dual-checkpoint inhibition. In this study, 135 patients with MGMT-silenced, microsatellite-stable metastatic CRC received two cycles of TMZ; those exhibiting disease stability (24% of the cohort) then entered a chemoimmunotherapy phase comprising low-dose ipilimumab plus nivolumab in conjunction with ongoing TMZ. Among the 33 patients treated in the combination phase, the objective response rate reached 45%, with a median progression-free survival of 7.0 months and overall survival of 18.4 months—substantially outperforming historical benchmarks for this population [73]. Moreover, serial monitoring of the TMZ-specific mutational signature in ctDNA demonstrated its utility as a noninvasive biomarker to guide the timing of immunotherapy initiation.

Collectively, these trials establish a framework in which TMZ-induced MMR deficiency and hypermutation can be harnessed to overcome primary resistance to ICI in pMMR CRC. The differential outcomes between single-agent PD-1 blockade in ARETHUSA and dual-checkpoint therapy in MAYA further suggest that a combination of CTLA-4 and PD-1 inhibition may be required to fully exploit the neoantigen repertoire generated by pharmacologically induced genomic instability. These clinical data, coupled with ongoing translational efforts, underscore the promise of targeted MMR disruption as a versatile strategy to sensitize colorectal tumors to immunotherapy.

## 8. MMR Deficiency in Early-Stage vs. Advanced Cancer

The problem of dMMR in early-stage versus advanced-stage cancers remains unresolved and is connected to the specific type of cancer being discussed. In 2019, the researchers identified patients from the Hellenic Cooperative Oncology Group (HeCOG)’s tumor repository with pMMR and dMMR nonmetastatic and colorectal cancer.

It was found that in patients with colorectal cancer, dMMR tumors were more likely to be a high grade (31% dMMR vs. 15% pMMR), and they were associated with lower stage disease (stage I/II) 63% of dMMR vs. 39% of pMMR. When it comes to the patients with EC, dMMR tumors were more likely to be low grade (80% of dMMR vs. 69% of pMMR). What is more, dMMR EC was positively associated with overall survival (HR = 0.38, 95% CI 0.20 to 0.76), which was not the case in patients with dMMR colorectal cancer (HR = 0.73, 95% CI 0.49 to 1.09) [74].

In 2023, another study comparing dMMR between early- and advanced-stage EC was conducted. The patients were divided based on MMR status and cancer stage. dMMR was observed in 43.9% of early-stage and 46.2% of advanced-stage EC. The researchers found no statistically significant differences in any of the groups in progression-free survival (83.9% in the pMMR group and 83.5% in the dMMR group) and in overall survival (OS) (83.9% in the pMMR group and 83.5% in the dMMR group). However, it was found that dMMR were more likely to invade myometrium (OD 2.35, 95% CI 1.21 to 4.57) [75].

On the contrary, in the same year, it was stated that MMR status is an effective prognostic factor in early-stage EC. Compared to pMMR, dMMR patients had worse two-year disease-free survival (93.0% in pMMR group vs. 86.8% in dMMR group) and two-year distant metastasis-free survival (96.3% in pMMR group vs. 95.0% in dMMR group). However, OS did not differ statistically between pMMR and dMMR patients (98.9% vs. 98.4%, respectively) [76].

A comprehensive overview of the key clinical trials investigating MMR-deficiency and immunotherapy across multiple cancer types is presented in Table 3

## 9. Conclusions and Future Perspectives

MMR is a well-established mechanism involved in maintaining genomic stability. Beyond its canonical role in correcting DNA replication errors, emerging evidence highlights its involvement in other biological processes, such as modulating immune responses and influencing tumor progression. These non-canonical functions underline the role of MMR in cancer biology, particularly in less common cancers.

Studies about MMR status in tumors not typically associated with its defects, such as neuroendocrine tumors, sarcomas, and hematologic malignancies, suggest that MMR may have diagnostic and prognostic relevance. In particular, studies have indicated that MMR could serve as a potential biomarker in SS. However, further MMR research in these rare cancers is crucial for creating novel diagnostic and therapeutic opportunities.

Innovative diagnostic techniques, including functional assays, liquid biopsy, and ctDNA analysis, are improving the identification of MMR defects and their biological consequences. Functional assays allow for a detailed understanding of MMR activity, while liquid biopsy and ctDNA provide minimally invasive methods to monitor tumor characteristics and treatment response in real-time. These advancements have significant implications for clinical decision-making, particularly in cases where traditional biopsies are difficult or insufficient.

Therapeutically induced MMR deficiency and naturally occurring dMMR both converge on the same fundamental principle: a hypermutated, neoantigen-rich tumor microenvironment that is exquisitely sensitive to immune checkpoint blockade. In colorectal cancer, transient MMR disruption via TMZ in MGMT-silenced, pMMR tumors (as shown in ARETHUSA and MAYA) successfully generates an acquired hypermutator phenotype and enables durable PD-1/CTLA-4–mediated control [67,73], mirroring the high immunogenicity—and favorable immunotherapy responses—seen in inherently dMMR early-stage disease. However, the prognostic and therapeutic impact of endogenous MMR loss varies with tumor stage and histology: while localized dMMR colorectal and EC often portend better outcomes, this benefit diminishes in advanced settings [74,75,76]. Together, these observations suggest that both engineered and natural MMR defects can be harnessed or anticipated to optimize immunotherapy, provided that patient selection, timing, and combination strategies are carefully tailored to each cancer’s clinical and molecular context.

MMR deficiency—whether arising endogenously through mutations in MLH1, MSH2, MSH6, or PMS2, or induced therapeutically—converges on a common biological consequence: a hypermutated tumor genome that generates abundant neoantigens, thereby rendering tumors susceptible to immune checkpoint blockade. The extensions of the clinical implications of MMR beyond colorectal and EC has shown that even understudied malignancies—neuroendocrine tumors, sarcomas, and select lymphomas—harbor MMR defects or high TMB that can guide immunotherapy selection. Moreover, it has been underscored that the prognostic value of MMR deficiency differs by cancer stage and histology: while early-stage dMMR colorectal and EC often predict favorable outcomes and ICI sensitivity, this advantage diminishes in advanced disease unless MMR loss is therapeutically imposed. Together, these insights advocate for a unified paradigm in which both intrinsic and engineered MMR defects serve as levers to amplify tumor immunogenicity.

Future efforts should focus on refining biomarker-driven patient selection (e.g., MGMT methylation, emergent MSH6 mutations, ctDNA-monitored mutational signatures), optimizing combination regimens (dual-checkpoint versus monotherapy), and extending MMR-altering strategies to a broader spectrum of “cold” tumors. By integrating molecular mechanisms with clinical experience, the field can tailor immunotherapy approaches that harness MMR dynamics to maximize durable anti-tumor immunity.

## Figures and Tables

**Table 1:** Proteins of the DNA mismatch repair pathway, their functions, and associated complexes.

Protein	Function	Complex
MSH2	Scaffold protein in MutSα/β	MutSα/MutSβ
MSH6	Recognizes base mismatches	MutSα
MSH3	Recognizes insertion–deletion loops	MutSβ
MLH1	Core component of MutL complexes	MutLα, MutLβ, MutLγ
PMS2	Endonuclease activity	MutLα
EXO1	Exonuclease for mismatch excision	N/A
PCNA	Coordinates DNA resynthesis	N/A
RPA	Protects single-stranded DNA	N/A
DNA Pol δ	DNA resynthesis	N/A
DNA Ligase I	DNA nicks ligation	N/A

**Table 2:** Diagnostic workflow and therapeutic implications of MMR/MSI status in cancer.

Diagnostic Step	Methods	Possible Results	Clinical Consequences	Therapeutic Implications
1. Initial MMR/MSI testing	IHC for MMR proteins (MLH1, MSH2, MSH6, PMS2); PCR-based MSI panels (e.g., pentaplex); targeted NGS panels that report MSI and TMB; ctDNA assays (liquid biopsy)	dMMR/MSI-H (loss of MMR protein expression or MSI detected)	High tumor mutational burden (often), increased neoantigen load and lymphocyte infiltration → higher probability of benefit from immune checkpoint inhibitors (ICI)	ICI are preferred/considered (e.g., pembrolizumab, nivolumab, dostarlimab) for eligible patients; consider clinical trials for combination strategies (dual checkpoint blockade, ATRi + ICI)
		pMMR/MSS (intact MMR protein expression; MSS on MSI assay)	Lower immunogenicity on average; less likely to respond to single-agent ICI	Standard systemic therapy (chemotherapy ± targeted agents according to tumour type and molecular profile); consider experimental sensitization strategies (e.g., TMZ priming, ATRi + ICI) within trials
2. Interpret discordant or borderline results	Re-test with complementary method (IHC ↔ PCR/NGS); evaluate sample quality; test metastatic site if clinically warranted	Heterogeneous/subclonal MMR or discordant results between assays	Potential for under- or over-estimation of dMMR; impacts treatment choice and trial eligibility	Confirmatory testing recommended before committing to long-term ICI; multidisciplinary tumour board discussion advised
3. Special/adjunct testing	Germline testing for MMR genes (MLH1, MSH2, MSH6, PMS2, EPCAM) if suspicion of Lynch/CMMRD; MGMT methylation in TMZ contexts; functional assays or ctDNA serial monitoring	Lynch syndrome/CMMRD; MGMT methylation status; emerging MMR mutations in ctDNA (post-treatment)	Hereditary cancer risk identified → implications for surveillance and family testing; MGMT status may predict TMZ effects; ctDNA may detect emergent resistance	Offer genetic counseling and cascade testing for relatives; use MGMT and ctDNA to guide experimental approaches (e.g., TMZ priming → consider trial enrollment)
4. Monitoring & follow-up	Serial imaging + ctDNA kinetics (when available)	Changes in ctDNA MSI/TMB signature or emergence of new MMR mutations	Early indication of therapy response or acquired resistance	Use ctDNA trends to time reassessment and consider switching/adding therapies or trial inclusion

Practical clarifications: 1. Methods note: For many tumour types (colorectal, endometrial) IHC and PCR/MSI panels remain gold standards; NGS panels provide complementary information (MSI + TMB + actionable mutations). Choose tests based on tumour type, available material, and local laboratory validation. 2. ctDNA note: Liquid biopsy (ctDNA) is useful when tissue is unavailable or for serial monitoring, but sensitivity varies with tumour shedding—a negative ctDNA MSI result does not reliably exclude tissue-confirmed dMMR; confirm on tissue when possible. 3. Discordance/heterogeneity: MMR status can be heterogeneous between primary tumour and metastases. Discordant IHC vs. molecular results should prompt repeat testing or testing of metastatic site; multidisciplinary review is recommended. 4. TMZ/experimental strategies: Temozolomide (TMZ) priming can induce hypermutation/emergent MSH6 mutations in some settings (e.g., MGMT-silenced CRC) and may sensitize to ICI; this approach remains experimental and is best used after multidisciplinary discussion. 5. TMB thresholds: TMB cutoffs differ by platform and tumour context; 6. Clinical caveat: Not all dMMR/MSI-H tumours will respond to ICI—consider additional factors (PD-L1, TMB, immune microenvironment, prior treatments) and clinical trial availability.

**Table 3:** Selected clinical trials of immunotherapy in mismatch repair–deficient (dMMR) cancers, organized by cancer type. Trials include both published studies and notable ongoing investigations.

Cancer Type	Trial & Agents (Phase)	NCT Number	Outcomes	Biomarker Criteria
Colorectal (mCRC)—Refractory (post-chemo)	CheckMate-142 (Phase II) Nivolumab ± Ipilimumab	NCT02060188	ORR ~49% with combo; 1-yr OS ~85%; durable control in ~83%	dMMR (MSI-H) selected
Colorectal (mCRC)—First Line	KEYNOTE-177 (Phase III) Pembrolizumab vs. chemo	NCT02563002	PFS 16.5 vs. 8.2 mo (HR = 0.60); ORR 44% vs. 33%; lower grade ≥ 3 AEs	dMMR (MSI-H) selected
Colorectal— Neoadjuvant (localized-colon)	NICHE (Phase II) Nivolumab + Ipilimumab	NCT03026140	100% pathologic response; 60% pCR in dMMR; some activity in pMMR	dMMR (MSI-H) tumors
Colorectal— Neoadjuvant (localized-rectal)	MSK Rectal trial (Phase II) Dostarlimab	NCT04165772	100% clinical CR in 14/14; no surgeries required at 6–12 mo follow-up	dMMR (MSI-H) tumors
Endometrial— Recurrent/Advanced	KEYNOTE-158 (Phase II) Pembrolizumab	NCT02628067	ORR ~45% (16% CR); median DOR ~47 mo	dMMR (MSI-H) selected
Endometrial—First Line	RUBY (Phase III) Dostarlimab + CT vs. CT	NCT03981796	PFS HR ~0.3 in dMMR; 2-yr OS 71% vs. 56%; OS benefit overall	Stratified by MMR status *(largest benefit in dMMR)*
Gastric/GE Junction—Advanced (post-chemo)	KEYNOTE-059 (Phase II) Pembrolizumab	NCT02335411	ORR 57.1% in MSI-H vs. ~9% in MSS; median DoR 16.2 mo	MSI-H subgroup analysis
Gastric/GE—First Line/Subgroup	KEYNOTE-062 (Phase III) Pembrolizumab ± CT	NCT02494583	ORR ~57% in MSI-H subgroup; trend toward improved OS/PFS	MSI-H subgroup analysis
Pan-Cancer (multiple types)—Refractory solid tumors	KEYNOTE-158 (Phase II) Pembrolizumab	NCT02628067	ORR 30.8%; median PFS ~4.2 mo; median OS ~23.5 mo; DOR ~47.5 mo	dMMR or MSI-H
Multiple (MSS tumors)—ATR inhibitor + IO	Phase II Ceralasertib (ATRi) + Durvalumab (PD-L1)	NCT03770494	ORR 22.6% in MSS mCRC (vs. ~0–5% with IO alone)	MSS (pMMR) tumors

dMMR—deficient mismatch repair (MMR-deficient); pMMR—proficient MMR (MMR-intact); MSI-H—microsatellite instability-high; MSS—microsatellite stable; TMB—tumor mutational burden (number of mutations per megabase of DNA; high TMB often overlaps with MSI-H); PD-1/PD-L1—programmed cell death-1/its ligand; CTLA-4—cytotoxic T-lymphocyte–associated antigen 4; ATR—Ataxia telangiectasia and Rad3–related kinase; ORR—objective response rate (percentage of patients with tumor size reduction ≥ a defined threshold); CR/PR—complete/partial response; DCR—disease control rate (CR + PR + stable disease); PFS—progression-free survival; OS—overall survival; HR—hazard ratio; EC—endometrial cancer; CRC—colorectal cancer; GE—gastroesophageal; IO—immunotherapy; AE—adverse event.

## Data Availability

Not applicable.

## Abbreviations

The following abbreviations are used in this manuscript:

ADDM	active DNA demethylation;
AE	adverse event;
AID	activation-induced deaminase;
ATR	ataxia telangiectasia and Rad3–related kinase;
BER	base excision repair;
cHL	classical Hodgkin Lymphoma;
CMMRD	constitutional mismatch repair deficiency;
CR/PR	complete/partial response;
CRC	colorectal cancer;
ctDNA	circulating tumor DNA;
CTLA-4	cytotoxic T-lymphocyte–associated antigen 4;
DCR	disease control rate (CR + PR + stable disease);
DDM	DNA demethylation;
DLBCL	diffuse large B-cell lymphoma;
dMMR	deficient mismatch repair (MMRdeficient);
dPCR	digital polymerase chain reaction;
EC	endometrial cancer;
EXO1	exonuclease 1;
GE	gastroesophageal;
HeCOG	hellenic cooperative oncology group;
HR	hazard ratio;
ICI	immune checkpoint inhibitors;
IDL	insertion-deletion loops;
IHC	Immunohistochemistry;
IO	immunotherapy;
LS	lynch syndrome;
MAF	mutant allele frequencies;
mCRC	metastatic colorectal cancers;
MEF	mouse embryonic fibroblasts;
MGMT	methylguanine DNA methyltransferase;
MLH	MutL homolog;
MMR	mismatch repair;
MSH	MutS homolog;
MSI	microsatellite instability;
MSI-H	microsatellite instability high;
MSS	microsatellite stable;
MutLα	MLH1-PMS2;
MutSα	MSH2-MSH6;
NEC	Neuroendocrine Cancer;
NET	neuroendocrine tumors;
NGS	next-generation sequencing;
ORR	objective response rate;
OS	overall survival;
PCNA	proliferating cell nuclear antigen;
PCR	polymerase chain reaction;
PD-1/PD-L1	programmed cell death-1/its ligand;
PFS	progression-free survival
PINK1	PTEN-induced putative kinase 1;
PMBCL	primary mediastinal B-cell lymphoma;
pMMR	proficient MMR (MMR-intact);
POLβ	polymerase beta;
POLγ	polymerase gamma;
ROS	reactive oxygen species;
RPA	replication protein A;
SCE	sister chromatid exchange;
SHM	somatic hypermutation;
SS	synovial sarcomas;
TLS	post-translesion synthesis;
TMB	tumor mutational burden;
TMZ	Temozolomide;
UV	ultraviolet;
VUS	variants of unknown significance;

## References

[1] Olave M.C., Graham R.P. (2022). Mismatch Repair Deficiency: The What, How and Why It Is Important. Genes Chromosomes Cancer.

[2] Rahimian E., Amini A., Alikarami F., Pezeshki S.M.S., Saki N., Safa M. (2020). DNA Repair Pathways as Guardians of the Genome: Therapeutic Potential and Possible Prognostic Role in Hematologic Neoplasms. DNA Repair.

[3] Amemiya K., Hirotsu Y., Nagakubo Y., Watanabe S., Amemiya S., Mochizuki H., Oyama T., Kondo T., Omata M. (2022). Simple IHC Reveals Complex MMR Alternations than PCR Assays: Validation by LCM and next-Generation Sequencing. Cancer Med..

[4] Grillo F., Angerilli V., Parente P., Vanoli A., Luchini C., Sciallero S., Puccini A., Bergamo F., Lonardi S., Valeri N. (2024). Prevalence and Type of MMR Expression Heterogeneity in Colorectal Adenocarcinoma: Therapeutic Implications and Reporting. Virchows Arch..

[5] Buchler T. (2022). Microsatellite Instability and Metastatic Colorectal Cancer—A Clinical Perspective. Front. Oncol..

[6] Moss E.L., Gorsia D.N., Collins A., Sandhu P., Foreman N., Gore A., Wood J., Kent C., Silcock L., Guttery D.S. (2020). Utility of Circulating Tumor DNA for Detection and Monitoring of Endometrial Cancer Recurrence and Progression. Cancers.

[7] Martín-Broto J., Moura D.S., van Tine B.A. (2020). Facts and Hopes in Immunotherapy of Soft-Tissue Sarcomas. Clin. Cancer Res..

[8] Germano G., Amirouchene-Angelozzi N., Rospo G., Bardelli A. (2018). The Clinical Impact of the Genomic Landscape of Mismatch Repair–Deficient Cancers. Cancer Discov..

[9] Martin S.A., Lord C.J., Ashworth A. (2010). Therapeutic Targeting of the DNA Mismatch Repair Pathway. Clin. Cancer Res..

[10] Guillotin D., Martin S.A. (2014). Exploiting DNA Mismatch Repair Deficiency as a Therapeutic Strategy. Exp. Cell Res..

[11] Brierley D.J., Martin S.A. (2013). Oxidative Stress and the DNA Mismatch Repair Pathway. Antioxid. Redox Signal..

[12] Martin S.A., McCabe N., Mullarkey M., Cummins R., Burgess D.J., Nakabeppu Y., Oka S., Kay E., Lord C.J., Ashworth A. (2010). DNA Polymerases as Potential Therapeutic Targets for Cancers Deficient in the DNA Mismatch Repair Proteins MSH2 or MLH1. Cancer Cell.

[13] Martin S.A., Hewish M., Sims D., Lord C.J., Ashworth A. (2011). Parallel High-Throughput RNA Interference Screens Identify PINK1 as a Potential Therapeutic Target for the Treatment of DNA Mismatch Repair-Deficient Cancers. Cancer Res..

[14] Santos F., Peat J., Burgess H., Rada C., Reik W., Dean W. (2013). Active Demethylation in Mouse Zygotes Involves Cytosine Deamination and Base Excision Repair. Epigenetics Chromatin.

[15] Franchini D.M., Chan C.F., Morgan H., Incorvaia E., Rangam G., Dean W., Santos F., Reik W., Petersen-Mahrt S.K. (2014). Processive DNA Demethylation via DNA Deaminase-Induced Lesion Resolution. PLoS ONE.

[16] Grin I., Ishchenko A.A. (2016). An Interplay of the Base Excision Repair and Mismatch Repair Pathways in Active DNA Demethylation. Nucleic Acids Res..

[17] Ijsselsteijn R., Jansen J.G., de Wind N. (2020). DNA Mismatch Repair-Dependent DNA Damage Responses and Cancer. DNA Repair.

[18] Tsaalbi-Shtylik A., Ferrás C., Pauw B., Hendriks G., Temviriyanukul P., Carlée L., Calléja F., Van Hees S., Akagi J.I., Iwai S. (2015). Excision of Translesion Synthesis Errors Orchestrates Responses to Helix-Distorting DNA Lesions. J. Cell Biol..

[19] Wada-Hiraike O., Yano T., Nei T., Matsumoto Y., Nagasaka K., Takizawa S., Oishi H., Arimoto T., Nakagawa S., Yasugi T. (2005). The DNA Mismatch Repair Gene HMSH2 Is a Potent Coactivator of Oestrogen Receptor α. Br. J. Cancer.

[20] Sajjadi E., Venetis K., Piciotti R., Invernizzi M., Guerini-Rocco E., Haricharan S., Fusco N. (2021). Mismatch Repair-Deficient Hormone Receptor-Positive Breast Cancers: Biology and Pathological Characterization. Cancer Cell Int..

[21] DeWitt J.T., Raghunathan M., Haricharan S. (2024). Nonrepair Functions of DNA Mismatch Repair Proteins: New Avenues for Precision Oncology. Trends Cancer.

[22] Drabløs F., Feyzi E., Aas P.A., Vaagbø C.B., Kavli B., Bratlie M.S., Peña-Diaz J., Otterlei M., Slupphaug G., Krokan H.E. (2004). Alkylation Damage in DNA and RNA—Repair Mechanisms and Medical Significance. DNA Repair.

[23] Uechi Y., Fujikane R., Morita S., Tamaoki S., Hidaka M. (2024). Bloom Syndrome DNA Helicase Mitigates Mismatch Repair-Dependent Apoptosis. Biochem. Biophys. Res. Commun..

[24] Robinson H.M.R., Black E.J., Brown R., Gillespie D.A.F. (2007). DNA Mismatch Repair and Chk1-Dependent Centrosome Amplification in Response to DNA Alkylation Damage. Cell Cycle.

[25] Dondi G., Coluccelli S., De Leo A., Ferrari S., Gruppioni E., Bovicelli A., Godino L., Coadă C.A., Morganti A.G., Giordano A. (2020). An Analysis of Clinical, Surgical, Pathological and Molecular Characteristics of Endometrial Cancer According to Mismatch Repair Status. A Multidisciplinary Approach. Int. J. Mol. Sci..

[26] Riedinger C.J., Esnakula A., Haight P.J., Suarez A.A., Chen W., Gillespie J., Villacres A., Chassen A., Cohn D.E., Goodfellow P.J. (2024). Characterization of Mismatch-Repair/Microsatellite Instability-Discordant Endometrial Cancers. Cancer.

[27] Huang Q., Yu T., Li L., Zhang Q., Zhang S., Li B., Li X., Xiao W., Liu G. (2023). Intraindividual Tumor Heterogeneity of Mismatch Repair Status in Metastatic Colorectal Cancer. Appl. Immunohistochem. Mol. Morphol..

[28] Marinelli D., Sabatini A., Bengala E., Ciurluini F., Picone V., Santini D., Pietrantonio F., Rossini D., Cremolini C. (2024). Systemic Treatment of Mismatch Repair Deficient/Microsatellite Instability-High Metastatic Colorectal Cancer—Single versus Double Checkpoint Inhibition. ESMO Open.

[29] André T., Shiu K.-K., Kim T.W., Jensen B.V., Jensen L.H., Punt C., Smith D., Garcia-Carbonero R., Benavides M., Gibbs P. (2020). Pembrolizumab in Microsatellite-Instability–High Advanced Colorectal Cancer. N. Engl. J. Med..

[30] Franke A.J., Skelton W.P., Starr J.S., Parekh H., Lee J.J., Overman M.J., Allegra C., George T.J. (2019). Immunotherapy for Colorectal Cancer: A Review of Current and Novel Therapeutic Approaches. J. Natl. Cancer Inst..

[31] Strickler J.H., Willis J.A. Treatment of Metastatic Colorectal Cancer: ASCO Guideline. www.asco.org/gastrointestinal-cancer-guidelines.

[32] Emiloju O.E., Zhu M., Xie H., Jin Z., Sinicrope F.A., Hubbard J.M. (2023). Selecting Optimal First-Line Treatment for Microsatellite Stable and Non-Mutated RAS/BRAF Metastatic Colorectal Cancer. Curr. Treat. Options Oncol..

[33] Baris H.N., Barnes-Kedar I., Toledano H., Halpern M., Hershkovitz D., Lossos A., Lerer I., Peretz T., Kariv R., Cohen S. (2016). Constitutional Mismatch Repair Deficiency in Israel: High Proportion of Founder Mutations in MMR Genes and Consanguinity. Pediatr. Blood Cancer.

[34] Buecher B., Le Mentec M., Doz F., Bourdeaut F., Gauthier-Villars M., Stoppa-Lyonnet D., Colas C. (2019). Syndrome CMMRD (Déficience Constitutionnelle Des Gènes MMR): Bases Génétiques et Aspects Cliniques. Bull. Cancer.

[35] Wimmer K., Rosenbaum T., Messiaen L. (2017). Connections between Constitutional Mismatch Repair Deficiency Syndrome and Neurofibromatosis Type 1. Clin. Genet..

[36] Siju J., Sahu A., Bhattacharya K., Prasad M., Sarin R., Gupta T. (2024). Demystifying the Mystery of Genes: A Case Report on Constitutional Mismatch Repair Deficiency. Indian J. Radiol. Imaging.

[37] Carsote M., Turturea I.F., Turturea M.R., Valea A., Nistor C., Gheorghisan-Galateanu A.-A. (2023). Pathogenic Insights into DNA Mismatch Repair (MMR) Genes–Proteins and Microsatellite Instability: Focus on Adrenocortical Carcinoma and Beyond. Diagnostics.

[38] Multone E., La Rosa S., Sempoux C., Uccella S. (2024). PD-L1 Expression, Tumor-Infiltrating Lymphocytes, and Mismatch Repair Proteins Status in Digestive Neuroendocrine Neoplasms: Exploring Their Potential Role as Theragnostic and Prognostic Biomarkers. Virchows Arch..

[39] Ban X., Mo S., Lu Z., Jia C., Shao H., Chang X., Mao X., Zhang Y., Pang J., Zhang Y. (2022). Expression and Methylation Status of MMR and MGMT in Well-Differentiated Pancreatic Neuroendocrine Tumors and Potential Clinical Applications. Endocrine.

[40] Tanaka K., Suzuki K., Miyashita K., Wakasa K., Kawano M., Nakatsu Y., Tsumura H., Yoshida M.A., Oda S. (2022). Activation of Recombinational Repair in Ewing Sarcoma Cells Carrying EWS-FLI1 Fusion Gene by Chromosome Translocation. Sci. Rep..

[41] He M., Abro B., Kaushal M., Chen L., Chen T., Gondim M., Yan W., Neidich J., Dehner L.P., Pfeifer J.D. (2020). Tumor Mutation Burden and Checkpoint Immunotherapy Markers in Primary and Metastatic Synovial Sarcoma. Hum. Pathol..

[42] Tawbi H.A., Burgess M., Bolejack V., Van Tine B.A., Schuetze S.M., Hu J., D’Angelo S., Attia S., Riedel R.F., Priebat D.A. (2017). Pembrolizumab in Advanced Soft-Tissue Sarcoma and Bone Sarcoma (SARC028): A Multicentre, Two-Cohort, Single-Arm, Open-Label, Phase 2 Trial. Lancet Oncol..

[43] Wienand K., Chapuy B., Stewart C., Dunford A.J., Wu D., Kim J., Kamburov A., Wood T.R., Cader F.Z., Ducar M.D. (2019). Genomic Analyses of Flow-Sorted Hodgkin Reed-Sternberg Cells Reveal Complementary Mechanisms of Immune Evasion. Blood Adv..

[44] Tian T., Li J., Xue T., Yu B., Li X., Zhou X. (2020). Microsatellite Instability and Its Associations with the Clinicopathologic Characteristics of Diffuse Large B-Cell Lymphoma. Cancer Med..

[45] Jeong A.R., Ball E.D., Goodman A.M. (2020). Predicting Responses to Checkpoint Inhibitors in Lymphoma: Are We Up to the Standards of Solid Tumors?. Clin. Med. Insights Oncol..

[46] Miyashita K., Fujii K., Taguchi K., Shimokawa M., Yoshida M.A., Abe Y., Okamura J., Oda S., Uike N. (2017). A Specific Mode of Microsatellite Instability Is a Crucial Biomarker in Adult T-Cell Leukaemia/Lymphoma Patients. J. Cancer Res. Clin. Oncol..

[47] Gilson P., Merlin J.L., Harlé A. (2021). Detection of Microsatellite Instability: State of the Art and Future Applications in Circulating Tumour Dna (Ctdna). Cancers.

[48] Sanz-Garcia E., Zhao E., Bratman S.V., Siu L.L. (2022). Monitoring and Adapting Cancer Treatment Using Circulating Tumor DNA Kinetics: Current Research, Opportunities, and Challenges. Sci. Adv..

[49] Silveira A.B., Bidard F.C., Kasperek A., Melaabi S., Tanguy M.L., Rodrigues M., Bataillon G., Cabel L., Buecher B., Pierga J.Y. (2020). High-Accuracy Determination of Microsatellite Instability Compatible with Liquid Biopsies. Clin. Chem..

[50] Georgiadis A., Durham J.N., Keefer L.A., Bartlett B.R., Zielonka M., Murphy D., White J.R., Lu S., Verner E.L., Ruan F. (2019). Noninvasive Detection of Microsatellite Instabilit and High Tumor Mutation Burden in Cancer Patients Treated with PD-1 Blockade. Clin. Cancer Res..

[51] Tiwari A.K., Roy H.K., Lynch H.T. (2016). Lynch Syndrome in the 21st Century: Clinical Perspectives. QJM.

[52] Martín-López J.V., Fishel R. (2013). The Mechanism of Mismatch Repair and the Functional Analysis of Mismatch Repair Defects in Lynch Syndrome. Fam. Cancer.

[53] Boeri M., Signoroni S., Ciniselli C.M., Gariboldi M., Zanutto S., Rausa E., Segale M., Zanghì A., Ricci M.T., Verderio P. (2024). Detection of (Pre)Cancerous Colorectal Lesions in Lynch Syndrome Patients by Microsatellite Instability Liquid Biopsy. Cancer Gene Ther..

[54] Jones J.J., Jones K.L., Wong S.Q., Whittle J., Goode D., Nguyen H., Iaria J., Stylli S., Towner J., Pieters T. (2024). Plasma CtDNA Enables Early Detection of Temozolomide Resistance Mutations in Glioma. Neurooncol. Adv..

[55] Kang S.Y., Kim D.G., Ahn S., Ha S.Y., Jang K.T., Kim K.M. (2022). Comparative Analysis of Microsatellite Instability by Next-Generation Sequencing, MSI PCR and MMR Immunohistochemistry in 1942 Solid Cancers. Pathol. Res. Pract..

[56] Gilson P., Levy J., Rouyer M., Demange J., Husson M., Bonnet C., Salleron J., Leroux A., Merlin J.L., Harlé A. (2020). Evaluation of 3 Molecular-Based Assays for Microsatellite Instability Detection in Formalin-Fixed Tissues of Patients with Endometrial and Colorectal Cancers. Sci. Rep..

[57] Smithgall M.C., Remotti H., Hsiao S.J., Mansukhani M., Liu-Jarin X., Fernandes H. (2022). Investigation of Discrepant Mismatch Repair Immunohistochemistry and Microsatellite Instability Polymerase Chain Reaction Test Results for Gynecologic Cancers Using Next-Generation Sequencing. Hum. Pathol..

[58] Meyers M., Hwang A., Wagner M.W., Boothman D.A. (2004). Role of DNA Mismatch Repair in Apoptotic Responses to Therapeutic Agents. Environ. Mol. Mutagen..

[59] Di Pietro M., Marra G., Cejka P., Stojic L., Menigatti M., Cattaruzza M.S., Jiricny J. (2003). Mismatch Repair-Dependent Transcriptome Changes in Human Cells Treated with the Methylating Agent N-Methyl-N′-Nitro-N-Nitrosoguanidine. Cancer Res..

[60] Bouvet D., Bodo S., Munier A., Guillerm E., Bertrand R., Colas C., Duval A., Coulet F., Muleris M. (2019). Methylation Tolerance-Based Functional Assay to Assess Variants of Unknown Significance in the MLH1 and MSH2 Genes and Identify Patients With Lynch Syndrome. Gastroenterology.

[61] Guan J., Li G.-M. (2023). DNA Mismatch Repair in Cancer Immunotherapy. NAR Cancer.

[62] Lenz H.J., Van Cutsem E., Limon M.L., Wong K.Y.M., Hendlisz A., Aglietta M., García-Alfonso P., Neyns B., Luppi G., Cardin D.B. (2022). First-Line Nivolumab Plus Low-Dose Ipilimumab for Microsatellite Instability-High/ Mismatch Repair-Deficient Metastatic Colorectal Cancer: The Phase II CheckMate 142 Study. J. Clin. Oncol..

[63] Qu F., Wu S., Yu W. (2024). Progress of Immune Checkpoint Inhibitors Therapy for PMMR/MSS Metastatic Colorectal Cancer. Onco Targets Ther..

[64] Matthaios D., Balgkouranidou I., Neanidis K., Sofis A., Pikouli A., Romanidis K., Pappa A., Karamouzis M., Zygogianni A., Charalampidis C. (2024). Revisiting Temozolomide’s Role in Solid Tumors: Old Is Gold?. J. Cancer.

[65] Zhang J., FG Stevens M., D Bradshaw T. (2011). Temozolomide: Mechanisms of Action, Repair and Resistance. Curr. Mol. Pharmacol..

[66] Germano G., Lamba S., Rospo G., Barault L., Magri A., Maione F., Russo M., Crisafulli G., Bartolini A., Lerda G. (2017). Inactivation of DNA Repair Triggers Neoantigen Generation and Impairs Tumour Growth. Nature.

[67] Crisafulli G., Sartore-Bianchi A., Lazzari L., Pietrantonio F., Amatu A., Macagno M., Barault L., Cassingena A., Bartolini A., Luraghi P. (2022). Temozolomide Treatment Alters Mismatch Repair and Boosts Mutational Burden in Tumor and Blood of Colorectal Cancer Patients. Cancer Discov..

[68] Sawada M., Hida T., Kamiya T., Minowa T., Kato J., Okura M., Idogawa M., Tokino T., Uhara H. (2024). Effects of Temozolomide on Tumor Mutation Burden and Microsatellite Instability in Melanoma Cells. J. Dermatol..

[69] Teng J.Y., Yang D.P., Tang C., Fang H.S., Sun H.Y., Xiang Y.N., Li X.M., Yang F., Xia R.X., Fan F. (2023). Targeting DNA Polymerase β Elicits Synthetic Lethality with Mismatch Repair Deficiency in Acute Lymphoblastic Leukemia. Leukemia.

[70] Saldanha J., Rageul J., Patel J.A., Kim H. (2023). The Adaptive Mechanisms and Checkpoint Responses to a Stressed DNA Replication Fork. Int. J. Mol. Sci..

[71] Wang M., Ran X., Leung W., Kawale A., Saxena S., Ouyang J., Patel P.S., Dong Y., Yin T., Shu J. (2023). ATR Inhibition Induces Synthetic Lethality in Mismatch Repair-Deficient Cells and Augments Immunotherapy. Genes Dev..

[72] Liu C., Wang X., Qin W., Tu J., Li C., Zhao W., Ma L., Liu B., Qiu H., Yuan X. (2023). Combining Radiation and the ATR Inhibitor Berzosertib Activates STING Signaling and Enhances Immunotherapy via Inhibiting SHP1 Function in Colorectal Cancer. Cancer Commun..

[73] Morano F., Raimondi A., Pagani F., Lonardi S., Salvatore L., Cremolini C., Murgioni S., Randon G., Palermo F., Antonuzzo L. (2022). Temozolomide Followed by Combination With Low-Dose Ipilimumab and Nivolumab in Patients With Microsatellite-Stable, O 6-Methylguanine-DNA Methyltransferase-Silenced Metastatic Colorectal Cancer: The MAYA Trial. J. Clin. Oncol..

[74] Fountzilas E., Kotoula V., Pentheroudakis G., Manousou K., Polychronidou G., Vrettou E., Poulios C., Papadopoulou E., Raptou G., Pectasides E. (2019). Prognostic Implications of Mismatch Repair Deficiency in Patients with Nonmetastatic Colorectal and Endometrial Cancer. ESMO Open.

[75] Chaowiwatkun K., Trongwongsa T., Rodpenpear N., Nutthachote P. (2023). Comparison of Tissue Mismatch Repair Protein Deficiency between Early and Advanced-Stage Endometrial Cancer. Asian Pac. J. Cancer Prev..

[76] Wang W., Yan Z., Hou X., Ren K., Hu K., Zhang F. (2023). Mismatch Repair Status Is an Effective Prognostic Factor for Early-Stage Endometrial Carcinoma. Int. J. Radiat. Oncol. Biol. Phys..

